# Functional Roles of Adenosine A_2B_ and A_3_ Receptor Subtypes in the Bladder of Male Rats With A_2B_ Receptor Deletion or Bladder Outlet Obstruction

**DOI:** 10.1002/nau.70430

**Published:** 2026-08-31

**Authors:** Masahiro Kurobe, Ei-ichiro Takaoka, Kanako Matsuoka, Shingo Kimura, Anne M. Robertson, Edwin K. Jackson, Lori A. Birder, Minoru Miyazato, Naoki Yoshimura

**Affiliations:** 1 Department of Urology, University of Pittsburgh School of Medicine, Pittsburgh, Pennsylvania, USA; 2 Department of Systems Physiology, Graduate School of Medicine, University of the Ryukyus, Ginowan, Okinawa, Japan; 3 Department of Mechanical Engineering and Materials Science, University of Pittsburgh, Pittsburgh, Pennsylvania, USA; 4 Department of Bioengineering, University of Pittsburgh, Pittsburgh, Pennsylvania, USA; 5 Department of Pharmacology & Chemical Biology, University of Pittsburgh School of Medicine, Pittsburgh, Pennsylvania, USA; 6 Department of Medicine, University of Pittsburgh School of Medicine, Pittsburgh, Pennsylvania, USA

**Keywords:** adenosine A2B and A3 receptors, bladder outlet obstruction, bladder overactivity, male rats

## Abstract

**Purpose:**

To investigate the adenosine receptor (AR) mechanism underlying bladder outlet obstruction (BOO)-related bladder dysfunction, we examined the expression level of AR subtypes in bladder tissues, and the effects of AR subtypes A2B & A3 modulation on bladder activity using male BOO rats, and Dahl rats with A2B receptor deletion.

**Materials and Methods:**

BOO was induced by partial ligation of the proximal urethra in male Sprague-Dawley rats. At 4 weeks after BOO, conscious cystometrograms (CMG) were performed with the urethral ligature being intact. The effects of AR A3 agonist (2CL-IB-MECA) and AR A3 antagonist (MRS3777) were examined by intravesical administration. Furthermore, we examined the difference of bladder function of AR subtypes between AR A2B knockout (A2B-KO) and wild-type groups of male Dahl rats. mRNA levels of AR subtypes in bladder tissues were also analyzed.

**Results:**

BOO versus sham rats showed heavier bladder weights, higher contraction amplitudes, and increased numbers of non-voiding contractions (NVCs) during the filling phase. In the bladder mucosa – but not the detrusor - of BOO versus sham rats, mRNA expression levels of A2A and A3 AR were significantly increased, while A2B levels were significantly decreased. In BOO rats, an A3 antagonist (MRS 3777; 10 and 100 μM) had no effect on CMG parameters. However, a high dose of an A3 agonist (2CL-IB-MECA; 100 μM) significantly increased NVCs (*p* = 0.0034), an effect that was blocked by the A3 antagonist. Additionally, compared to wild-type rats, A2B-KO rats showed a significant increase in the number of NVCs (*p* = 0.0039).

**Conclusions:**

A2B AR down-regulation in BOO rats and A2B deletion in Dahl rats could contribute to the development of bladder overactivity. Moreover, a high-level of exogenous activation of A3 ARs might exacerbate bladder overactivity in BOO.

## 1 ∣ Introduction

Urinary frequency, urgency and urgency incontinence are common bothersome storage symptoms in men with partial bladder outlet obstruction (BOO) due to benign prostate hyperplasia (BPH) [1]. Bladder overactivity, characterized by involuntary bladder contractions during the filling phase of cystometry, is thought to be a major cause of storage symptoms in patients with BPH [1]. Animal models with BOO, caused by partial urethral obstruction, also have bladder overactivity, evidenced by non-voiding contractions (NVCs) during the filling phase of cystometry. NVCs are frequently observed in pathophysiological conditions, especially in animal models of BOO [2]. These NVCs are similar to the involuntary contractions seen in patients with BOO. It has been demonstrated that NVCs in BOO model animals were largely of myogenic origin [2], which may be strongly related with the function of the detrusor smooth muscles. Thus, BOO rats are widely considered to be an appropriate animal model for studying the causes of bladder overactivity associated with BPH [3, 4].

Adenosine, a breakdown product of adenosine triphosphate (ATP), can activate four different subtypes of G protein-coupled receptors (A1, A2A, A2B and A3), to elicit numerous physiological effects in various organs [5]. Adenosine can be produced and released by the urothelium, and all four receptor subtypes (A1, A2A, A2B, and A3) are expressed in the uroepithelium, as previously confirmed at the protein level [6]. Adenosine may function as an autocrine/paracrine regulator of exocytosis in the uroepithelial layer [6] and may play an important role in modulating sensory afferent function and smooth muscle contraction [7]. Previous studies including ours also showed that adenosine receptor (AR) subtypes are involved in the control of bladder function under normal and inflammatory conditions [8-10]. A recent study has also examined the subtype specific role of AR in BOO-induced bladder dysfunction, and found that mRNA expression levels of AR A2A were upregulated in the bladder mucosa and contribute to bladder overactivity after BOO [11]. Thus, this study further examined the effects of modulation of other subtypes, such as AR A2B & A3, on bladder activity using male BOO rats.

## 2 ∣ Materials and Methods

### 2.1 ∣ Animals

Male Sprague-Dawley rats (aged 16 weeks) were used to produce a BOO model and to examine the role of adenosine A3 receptors. Additionally, male, 16-week-old Dahl salt-sensitive (Dahl SS) rats and A2B receptor knockout rats in Dahl SS background were obtained from colonies at the University of Pittsburgh that were bred and maintained by Dr. Edwin K. Jackson's laboratory (University of Pittsburgh, PA, USA). These A2B knockout (A2B-KO) rats were generated by the MCW Gene Editing Rat Resource Program (Dr. Aron M. Geurts, Department of Physiology and Human Molecular Genetics Center, Medical College of Wisconsin, Milwaukee, WI) and were previously described and characterized by Jackson and coworkers [12]. Both groups of rats were housed in plastic cages with soft bedding and free access to food and water under a 12/12 h reversed light-dark cycle. Dahl SS rats were kept on a low salt diet (0.3% NaCl, Research Diets Inc. New Brunswick, NJ) to prevent hypertension, and deletion of A2B genes were verified by genomic DNA genotyping and expression analysis [12]. All experimental procedures were carried out in compliance with the ARRIVE and NIH guidelines and approved by the Institutional Animal Care and Use Committee (IACUC) at the University of Pittsburgh (Approval # 22122280).

### 2.2 ∣ A Rat Model of BOO

We used male Sprague-Dawley rats for producing the BOO model according to the methods previously described [13] with slight modification. Under isoflurane anesthesia (3%–5% concentration for induction followed by a reduction to 1%–3% for maintenance), the bladder and the proximal urethra were exposed via a lower abdominal midline incision. A 4-0 silk ligature was placed around the urethra and tied at the urethrovesical junction level proximal to the urethral fenestration with a metal rod (outside catheter diameter of 1.27 mm) placed alongside the urethra, and then the rod was removed. The abdominal wound was closed. This ligation was maintained in place throughout the duration of the experiments. Four weeks after inducing BOO, the rats were prepared for functional and molecular studies. In sham rats, we operated on the animals with similar procedures without urethral ligation.

### 2.3 ∣ Awake Continuous Cystometry

Cystometry (CMG) under an awake condition was performed as described previously [14]. Under isoflurane anesthesia (3%–5% concentration for induction followed by a reduction to 1%–3% for maintenance), a PE-50 polyethylene catheter (Clay-Adams, Parsippany, NJ) was inserted through the bladder dome, and a purse-string suture was placed tightly around the catheter. The implanted catheter was exteriorized through the abdominal wall, and the wound was closed with 4-0 silk sutures. After the surgery, the rats were placed in restraining cages (W 80 mm × L 300 mm × H 150 mm, Yamanaka Chemical Ind. Ltd, Osaka, Japan) and allowed to recover from isoflurane anesthesia for 1–2 h before starting CMG. The intravesical catheter was connected via a three-way stopcock to a pressure transducer (Transbridge 4 M, World Precision Instruments, Sarasota, FL, USA) for recording intravesical bladder pressure, and a syringe pump for infusing saline. Because variability in bladder capacity among BOO rats is typically observed in this model, saline was initially infused at 0.1 ml/min; subsequently, the infusion rate was adjusted to 0.04–0.3 mL/min to obtain a similar inter-contraction interval around 10–15 min among BOO animals [13]. In Dahl rats with or without A2B receptor deletion, saline was infused at 0.1 ml/min. Intravesical pressure changes were measured using data acquisition software (AD Instruments, Castle Hill, NSW, Australia) at a sampling rate of 100 Hz using a PowerLab. Saline infusion was continued until stable voiding cycles were established.

The following CMG parameters were evaluated:(i) amplitude (the peak pressure minus the basal pressure during each contraction period); (ii) baseline pressure, the pressure immediately after the reflex contraction; and (iii) threshold pressure, the pressure immediately before the reflex contraction. In addition, NVCs during the storage phase were defined as small-amplitude bladder contractions that occur without voiding with pressure elevation > 4 cmH_2_O from baseline pressure [13]. The average number of NVCs per minute between voiding contractions was determined. Obtained measurements for each rat represented an average of three to five bladder contractions after the initial 60 min stabilization period. Then, voided urine was collected and measured to determine voided volume, and post-void residual volume (PVR) was collected by gravity with the bladder catheter. Bladder capacity was calculated as the sum of voided and residual volumes. Voiding efficiency (VE) was also calculated as the ratio of voided volume divided by bladder capacity. Finally, bladder compliance was calculated as bladder capacity divided by the difference in threshold pressure and baseline pressure values in CMG, which were obtained after bladder emptying to eliminate the effect of PVR on bladder compliance. Some of BOO rats (*n* = 6) showed urinary retention during CMG, and these rats were excluded from further analyses.

### 2.4 ∣ Intravesical Administration of Adenosine A3 Receptor Acting Drugs

Other sets of male Sprague-Dawley rats with or without BOO were used for these experiments. Stock solutions of an A3 agonist (2CL-IB-MECA, 1-[2-Chloro-6-[[(3-iodophenyl)methyl]amino]-9H-purin-9-yl]-1-deoxy-N-methyl-β-d-ribofuranuronamide, CAS No. 163042-96-4, Tocris Bioscience, Minneapolis, MN) were made in 100% Dimethyl sulfoxide (DMSO), and these were further diluted with saline to a final concentration of 1% DMSO. After baseline saline CMG measurements were obtained, 10 μM of 2CL-IB-MECA was intravesically administered to BOO rats (*n* = 10). After 15 min of 2CL-IB-MECA infusion, CMG parameters were measured and compared to pre-drug baseline values. Then, we administrated 100 μM of 2CL-IB-MECA into the bladder of BOO rats. After 15 min, CMG parameters were measured. Drug doses were determined according to the results of pilot experiments as well as previous reports [15]. We also examined the effects of 2CL-IB-MECA at 10 μM and 100 μM in sham rats (*n* = 10) to confirm that these concentrations do not influence normal bladder function. Also, in sham rats (*n* = 10), it was confirmed that intravesical 1% DMSO vehicle does not affect bladder function.

We further examined the effects of an A3 antagonist (MRS3777 hemioxalate, N-cyclohexyl-2-phenoxy-7H-purin-6-amine; oxalic acid, CAS No. 1186195-57-2, Tocris Bioscience, Minneapolis, MN) in other sets of BOO rats and sham rats. Stock solutions of MRS3777 were made in 100% DMSO, and these were further diluted with saline to a final concentration of 1% DMSO. Drug doses were determined according to the results of pilot experiments as well as previous reports [16, 17]. We examined the effect of MRS3777 at 10 μM and 100 μM on sham rats (*n* = 10) to confirm that these concentrations do not influence normal bladder function. Then, we administrated MRS3777 at these concentrations into the bladder of BOO rats (*n* = 10).

Finally, we examined whether the effects of an A3 agonist (2CL-IB-MECA) are blocked by an A3 antagonist (MRS3777) in other set of BOO rats (*n* = 7). MRS3777 (100 μM) was first administered intravesically, and CMG parameters during two micturition cycles were measured and compared to baseline values. Then, intravesical co-administration of 2CL-IB-MECA (100 μM) and MRS3777 (100 μM) was performed, and CMG parameters during three micturition cycles were evaluated. All CMG recordings were completed within 3 h from the start of saline infusion in accordance with previous studies [11].

### 2.5 ∣ Quantification of AR Subtypes and GAPDH mRNA

To evaluate the change in mRNA levels of AR in bladder mucosa and detrusor layers, real-time PCR (RT-PCR) were performed in separate groups of rats. Bladder tissues were excised at the level of the bladder neck and cut longitudinally into halves from sham (*n* = 6) and BOO (*n* = 6) Sprague-Dawley rats as well as wild-type (*n* = 4) and A2B receptor knock-out Dahl SS rats (*n* = 3). These animals did not undergo CMG to avoid the influence of repeated bladder contractions during CMG on molecular transcript levels in the bladder. One half of the bladder tissue was then used for molecular analyses. The bladder specimens were separated into mucosal and detrusor layers using microscissors and stored at −80°C until mRNA analyses. Total RNA was extracted from the tissue using TRIzol reagent (Invitrogen, Carlsbad, CA). Five micrograms of RNA were reverse-transcribed into cDNA using Superscript II (Invitrogen). Gene expressions of AR subtypes such as A1 (ADORA1), A2A (ADORA2A), A2B (ADORA2B), and A3 (ADORA3) receptors were quantified with an MX3000P real-time PCR system (Stratagene, La Jolla, CA) in a 25 μL volume using SYBR Green PCR Master Mix9 (QIAGEN, Valencia, CA). Reactions were polymerase activation 95°C/15 min, denaturation by cycling 40 times at 94°C/15 s, annealing 55°C/30 s, and extension 72°C/30 s. The relative expression levels of each marker were normalized by a housekeeping gene (glyceraldehyde-3-phosphate dehydrogenase; GAPDH). Primer sequences used for RT-PCR are described in supplementary table (Table S1).

### 2.6 ∣ Statistical Analysis

The data are shown as the mean ± standard deviation (SD). Data were analyzed using JMP^®^ software (version 11; SAS Institute, Cary, NC). A one-way ANOVA followed by Dunnett's multiple comparison test was used for the statistical comparison among groups. Student's paired t-test was used for the comparison of data values before and after drug administration. *p*-values < 0.05 were considered to be statistically significant.

## 3 ∣ Results

### 3.1 ∣ The Effect of BOO in Male Sprague-Dawley Rats

Bladder weights were higher in BOO rats (*n* = 6, 362.0 ± 27.5 mg) compared to sham rats (n = 6, 123.4 ± 15.9 mg). Figure 1A shows representative tracings of awake CMG in sham and BOO rats. Contraction amplitudes were increased in BOO rats (81.02 ± 19.3 cmH_2_O) compared to sham rats (35.93 ± 7.54 cmH_2_O) (*p* = 0.0044, Figure 1A). The number of NVCs was also increased in BOO rats (0.950 ± 0.312/min) compared to sham rats (0.309 ± 0.121/min) (*p* = 0.0044, Figure 1B). On the other hand, bladder compliance was increased in BOO rats (0.283 ± 0.127 ml/cmH_2_O) compared to sham rats (0.0993 ± 0.038 ml/cmH_2_O, *p* = 0.0218). Bladder capacity was also increased in BOO rats (1.958 ± 0.576 ml) compared to sham rats (0.775 ± 0.307 ml, *p* = 0.0041). PVR was increased in BOO rats (0.620 ± 0.293 ml) compared to sham rats (0.003 ± 0.0075 ml, *p* = 0.0008). VE was decreased in BOO rats (66.88 ± 16.1%) versus sham rats (99.53 ± 1.04%, *p* = 0.0062).

### 3.2 ∣ The Effect of Intravesical Administration of Adenosine A3 Receptor Acting Drugs in Sprague-Dawley Rats

In male Sprague-Dawley rats, intravesical administration of 1% DMSO in sham or BOO rats did not influence bladder function. In sham rats, intravesical administration of 10-100 μM A3 agonist (2CL-IB-MECA) and 10–100 μM A3 antagonist (MRS3777) did not influence bladder function. Figure 2A shows representative CMG recordings in BOO rats during intravesical administration of 10 μM and 100 μM 2CL-IB-MECA. In BOO rats, intravesical administration of 100 μM 2CL-IB-MECA increased the number of NVCs compared to predrug baseline values (*p* = 0.0034), although there were no statistically significant effects on other CMG parameters (Table 1). Figure 2B shows representative CMG recordings in BOO rats during intravesical administration of 10 μM and 100 μM MRS3777. In BOO rats, intravesical administration of MRS3777 had no statistically significant effects on any CMG parameters (Table 1). Furthermore, in BOO rats, intravesical co-administration of 100 μM 2CL-IB-MECA following 100 μM MRS3777 administration did not induce statistically significant changes in CMG parameters (Figure 2C, Table 1), indicating that the facilitatory effects of 2CL-IB-MECA on BOO-induced bladder overactivity were counteracted by MRS3777, a selective adenosine A3 antagonist, in BOO rats.

### 3.3 ∣ The Effect of Adenosine A2B Receptor Deletion in Male Dahl Rats

There were no differences in bladder weight of A2B-KO rats (*n* = 3) and wild-type rats (*n* = 4) (127.3 ± 3.71 vs. 115.8 ± 6.50 mg, respectively) (Figure 3B). Figure 3A shows representative tracings of awake CMG in wild-type and A2B-KO Dahl rats. The number of NVCs were increased in A2B-KO rats (0.55 ± 0.43/min) compared to wild-type rats (0.13 ± 0.15/min) (*p* = 0.0039, Figure 3A, B). Bladder compliance was also increased in A2B-KO rats (0.087 ± 0.026 ml/cmH_2_O) compared to wild-type rats (0.053 ± 0.016 ml/cmH2O) (*p* = 0.0405, Figure 3B).

### 3.4 ∣ Molecular Analyses

In male Sprague-Dawley rats, there were no statistically significant differences for mRNA expression levels of AR subtypes in bladder muscle tissue between BOO rats and sham rats; however, mRNA levels of A2A and A3 were increased, and A2B levels were decreased in BOO mucosa versus sham (Figure 4A). In addition, the expression levels of GAPDH in the bladder mucosa was not statistically different between sham and BOO groups (Ct values: 24.2 ± 0.85 vs. 24.0 ± 1.07, *p* = 0.7449). In male Dahl rats with A2B receptor deletion, there were no statistically significant differences for mRNA expression levels of AR subtypes except A2B in bladder muscle tissues (Figure 4B). In the mucosal tissue, mRNA levels of A1 and A2A were increased in A2B-KO rats versus wild-type rats (Figure 4B). In addition, the expression levels of GAPDH in the bladder mucosa were not statistically different between wild-type and A2B-KO groups (Ct values: 24.18 ± 0.93 vs. 24.6 ± 0.97, *p* = 0.6418). The expression levels of GAPDH in the bladder muscle were not statistically different either between in wild-type and A2B-KO groups (Ct values: 27.0 ± 1.48 vs. 27.1 ± 1.03, *p* = 0.9440).

## 4 ∣ Discussion

The results of this study indicate that; (1) BOO rats showed higher bladder weights, higher contraction amplitude, and more NVCs during the filling phase versus sham, (2) mRNA levels of AR subtypes A2A and A3, were increased while A2B levels were decreased in the bladder mucosa of BOO rats versus sham, (3) the number of NVCs were increased in A2B-KO Dahl rats versus wild-type rats, (4) mRNA levels of AR A1 and A2A subtypes were increased in the bladder mucosa of A2B-KO versus wild-type, (5) in sham rats, intravesical A3 agonist or antagonist administration did not influence bladder function, and (6) in BOO rats, intravesical administration of an A3 agonist increased the number of NVCs, and these effects were blocked by an A3 antagonist.

Therefore, based on these findings, it is likely that down-regulation of the mRNA level of AR A2B receptor subtype leads to bladder overactivity in BOO rats, while the AR A3 subtype does not play a predominant role in the emergence of bladder overactivity although exogenous, high-level activation of AR A3 receptors might exacerbate bladder overactivity in pathological status like BOO.

Animal models with BOO, caused by partial bladder outlet obstruction, exhibit bladder hypertrophy shown by wall thickness, resulting in an increase in bladder weight, similar to patients with BOO [18]. The degree of BOO in this study seems to be reasonably enough as the bladder weights of BOO rats, which were approximately three times heavier than the sham rats, were equivalent to those reported in previous studies [19, 20]. In addition, NVCs are also observed in BOO models [13, 21], which indicates bladder dysfunction such as bladder overactivity as similarly showed in patients with BPH [22]. Therefore, we evaluated cystometric parameters including NVCs as a surrogate marker of bladder overactivity. As in previous reports [13, 21], contraction amplitudes and NVCs were increased in BOO rats compared to sham rats, indicative of bladder overactivity after BOO. Although both sexes have been used as BOO rat models, the female rat has been used more commonly because of the simpler anatomy and the absence of the accessory sex organs. However, Zvara et al. reported that expression level of nerve growth factor mRNA in bladder tissues of BOO model rats were different between male and female [23]. Therefore, we used male rats for producing a BOO model, which is more physiologically relevant with the BOO condition in men with BPH.

Adenosine is produced by degrading ATP released from the urothelium and may play an important role in modulating sensory afferent function and smooth muscle contraction in the bladder [7]. The physiological effects of adenosine are known to be mediated by four different subtypes, such as A1, A2A, A2B and A3 receptors. The present study showed that, following BOO for 4 weeks, transcript levels of A2A and A3 receptors were upregulated and A2B receptors were downregulated in the bladder mucosa whereas A1 receptor levels were not statistically different in the bladder mucosa of BOO rats versus sham rats. A recent study using the same BOO model has shown similar results in terms of changes in mRNA levels of AR subtype expression, and that intravesical application of a selective A2A receptor antagonist (ZM241385) reduced NVCs during bladder filling in BOO rats, whereas A1 receptor activation by CCPA had no statistically significant effects on bladder activity in sham or BOO rats [11]. These data indicate that adenosine A2A receptor upregulation in the bladder contributes to the emergence of bladder overactivity in male BOO rats. The present study further elucidated the roles of down-regulated A2B and upregulated A3 receptors in bladder overactivity using transgenic rats with A2B receptor deletion and BOO model rats. Because we found that A2B receptor deletion in male Dahl rats exhibited bladder overactivity, evident as the increased number of NVCs during bladder filling, it is assumed that adenosine A2B receptors play an inhibitory role in the control of bladder function and that the reduced expression of bladder A2B receptors in the bladder mucosa could be involved in the emergence of bladder overactivity after BOO.

In previous studies, adenosine acting via A2B receptors has been implicated in the modulation of detrusor relaxation in bladder muscle strips from humans and guinea pigs [24, 25]. Also, in isolated tissue assays, an A2B agonist BAY60-6583 decreased the amplitude of spontaneous activity in bladder muscle strips from rats with spinal cord injury [15]. Decreases in expression of the A2B receptor have been reported to contribute to a reduction in evoked detrusor relaxation with aging [26]. Hao Y et al. also reported that A2B receptor KO mice have an overactive bladder phenotype [27]. These findings are therefore consistent with our studies emphasizing the importance of A2B receptor function for inhibition of bladder overactivity. Thus, adenosine A2B receptor activation could be a therapeutic modality for male patients with OAB due to BPH-induced BOO.

Next, the results of the present study revealed that the A3 receptor dose not play a dominant role in the emergence of bladder overactivity in BOO rats although the expression level of A3 receptors was increased in bladder mucosa after BOO (Figure 4A). In this study, intravesical administration of an A3 agonist or antagonist did not influence bladder function in sham rats. Furthermore, intravesically applied A3 antagonist (MRS3777) did not affect NVCs in BOO rats even though A3 receptor expression was upregulated in the bladder mucosa after BOO, indicating that A3 receptor upregulation does not play a dominant role in BOO-induced bladder overactivity. However, interestingly, intravesical administration of an A3 agonist at a higher concentration (100 μM) induced a statistically significant increase of the number of NVCs, and these effects were blocked by an A3 antagonist in BOO rats. Thus, it is assumed that exogenously applied, high-concentration A3 receptor agonist can induce an excessive effect on BOO-induced bladder activity in association with up-regulation of A3 receptor expression in the bladder mucosa of BOO rats. In addition, previous studies demonstrated that selective A3 receptor agonists were without notable effects on any contractile variables in isolated strips of the human stable bladder, the human neuropathic detrusor overactivity bladder or the guinea-pig bladder [24, 25]. In isolated tissue assays, inosine decreased the amplitude of spontaneous activity in bladder muscle strips from rats with spinal cord injury; however, this effect was not prevented by selective A3 antagonists [15]. Vesela R et al. also reported that an addition of A3 antagonists increased the adenosine-evoked relaxation in the presence of general P2 purinoceptor antagonists and A2A antagonists in bladder strips from both normal and cyclophosphamide pre-treated rats [28]. These results indicate that activation of adenosine A3 receptors induces contraction of the bladder in some specific conditions possibly with interactions with other adenosine receptors [29]. Taken together, while A3 receptor activation does not seem to play a dominant role in the control of bladder activity, exogenous and excessive activation of A3 receptors might exacerbate bladder overactivity in male BOO patients.

There are some limitations in this study. First, we did not perform pharmacological testing in A2B deletion rats; therefore, the possibility that A2A upregulation may contribute to bladder overactivity in A2B KO rats cannot be excluded. Further experiments will be needed to clarify this point. Second, we evaluated the role of subtypes of AR in bladder dysfunction in the early phase of BOO (4 weeks). The long-term BOO causes bladder decompensation, often associated with the underactive bladder condition with tissue fibrosis [30]. However, the role and distribution of adenosine receptors in chronic phase of BOO model have not been investigated. Thus, future studies are needed to investigate the long-term effects of BOO on adenosine receptor functions involved in BOO-induced bladder under-activity and/or tissue fibrosis.

## 5 ∣ Conclusions

In conclusion, down-regulation of adenosine A2B receptors could contribute to the development of bladder overactivity, and high-level, exogenous activation of A3 receptors might exacerbate bladder overactivity in BOO.

## Supplementary Material

Table S1

Additional supporting information can be found online in the Supporting Information section.

**Supporting File:** nau70430-sup-0001-Supplementally_Table_S1.docx.

## Figures and Tables

**FIGURE 1 ∣:**
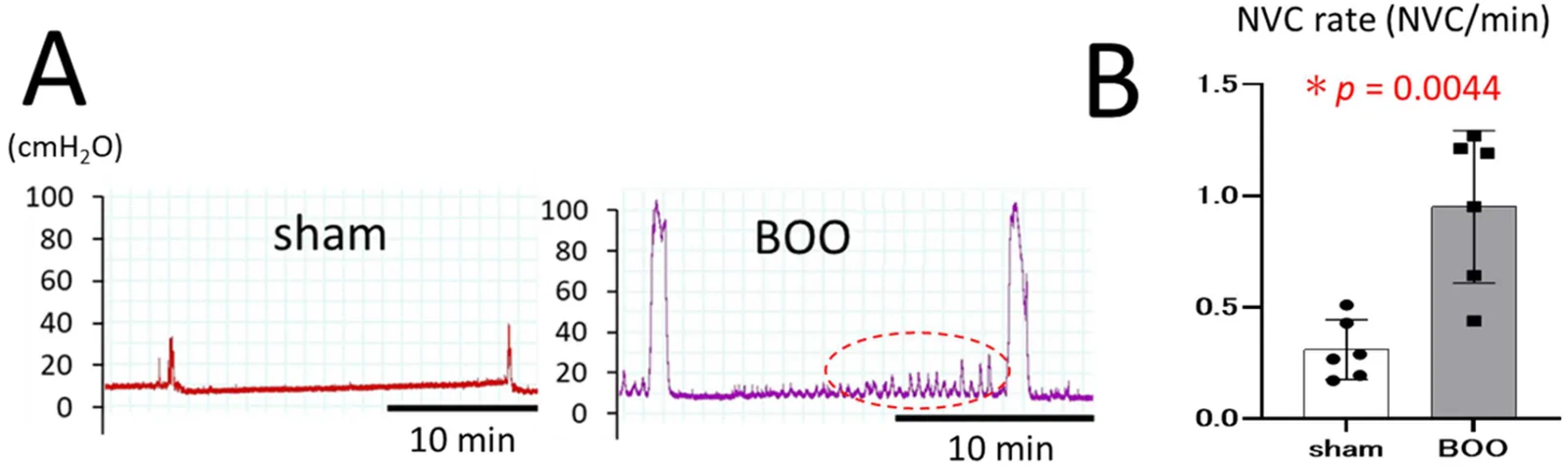
Effects of BOO in male Sprague-Dawley rats. (A) Representative CMG traces in sham and 4-weeks BOO rats. (B) Comparison of the number of NVCs during bladder filling.

**FIGURE 2 ∣:**
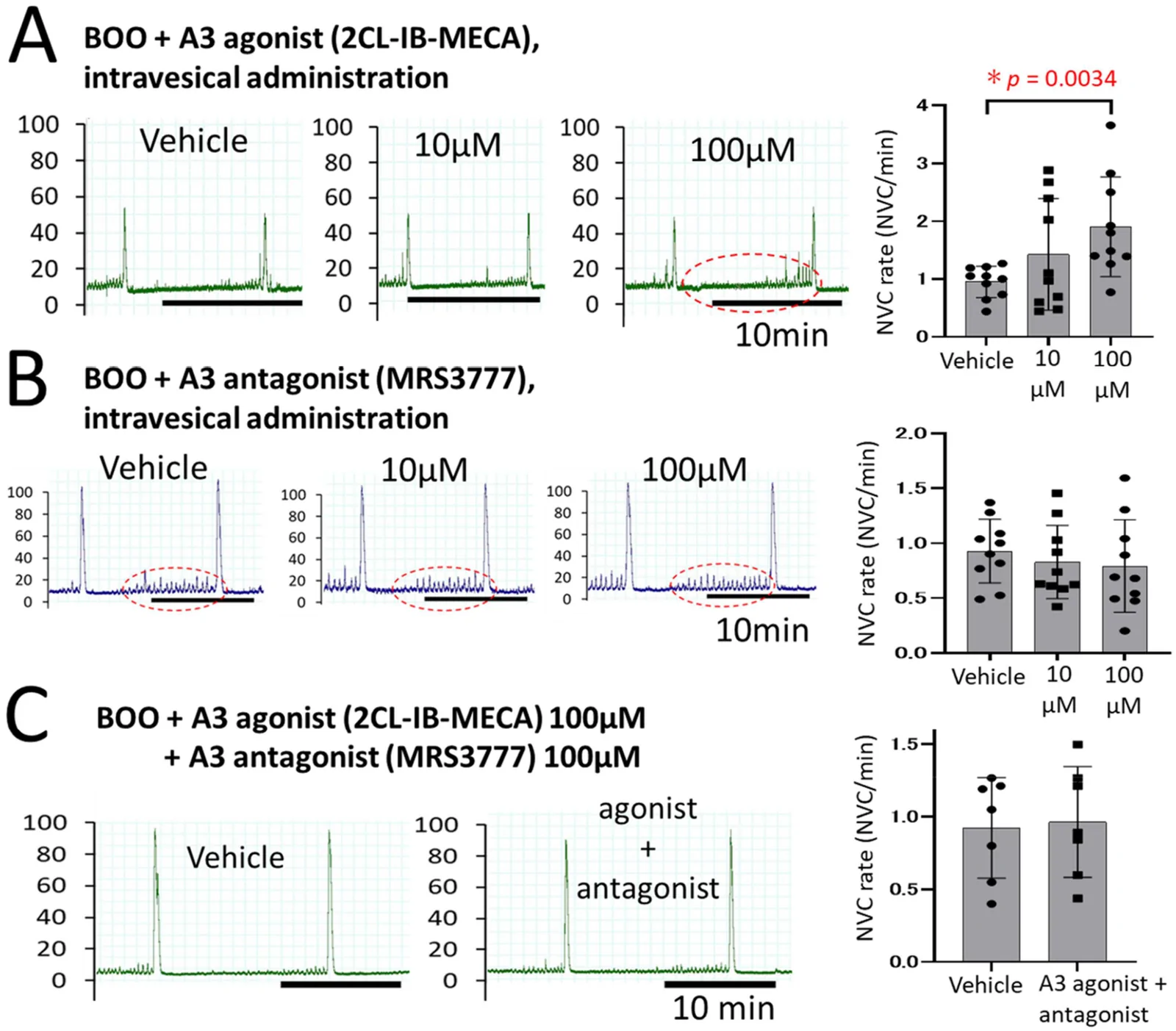
Effects of intravesical administration of adenosine A3 receptor acting drugs in BOO rats (A) Effects of an A3 agonist (2CL-IB-MECA). (B) Effects of an A3 antagonist (MRS3777). (C) Effects of co-administration of A3 agonist and antagonist.

**FIGURE 3 ∣:**
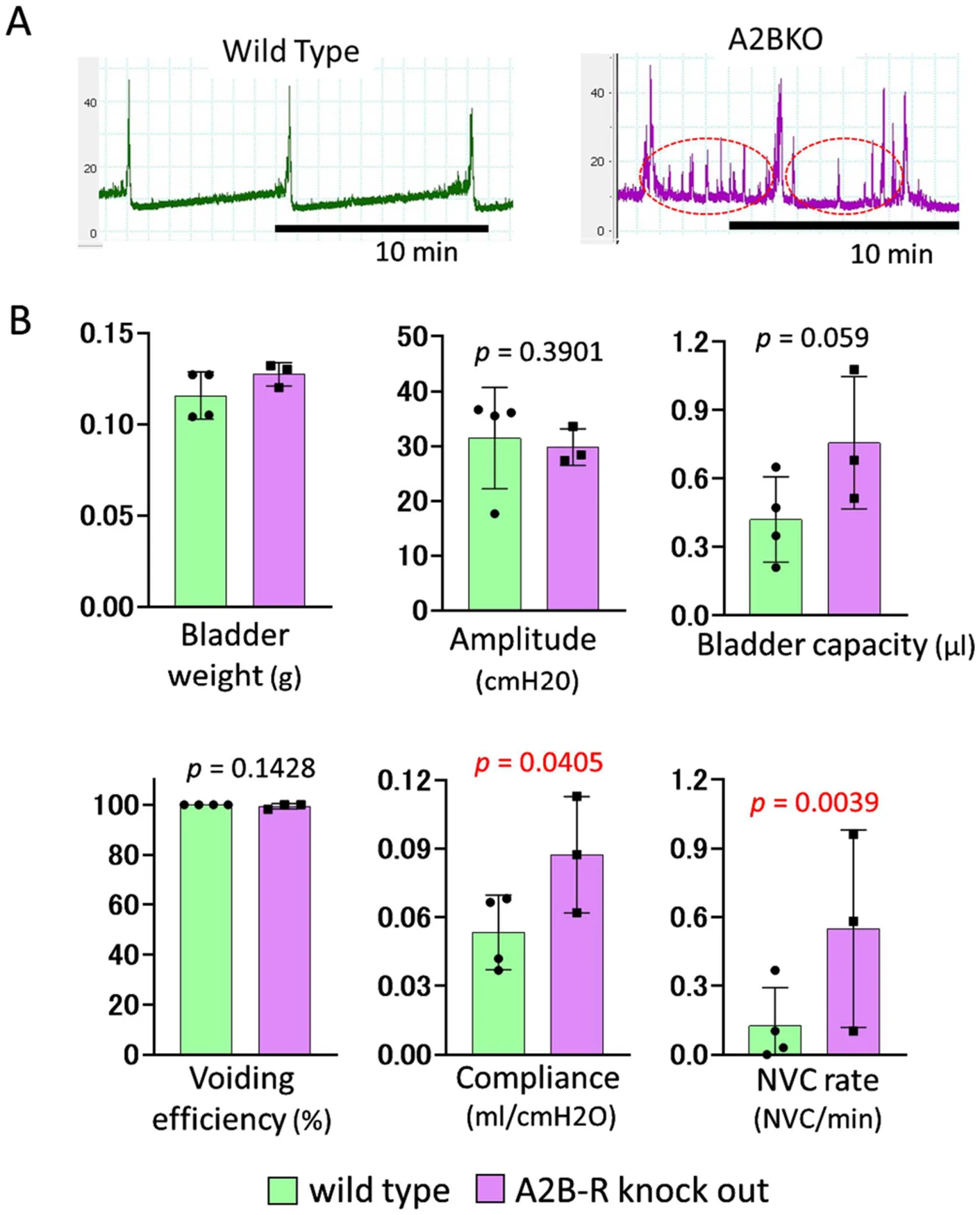
Effects of adenosine A2B receptor deletion in Dahl rats. (A) Representative CMG traces in wild-type and A2B knockout rats. (B) Comparison of bladder weights and CMG parameters.

**FIGURE 4 ∣:**
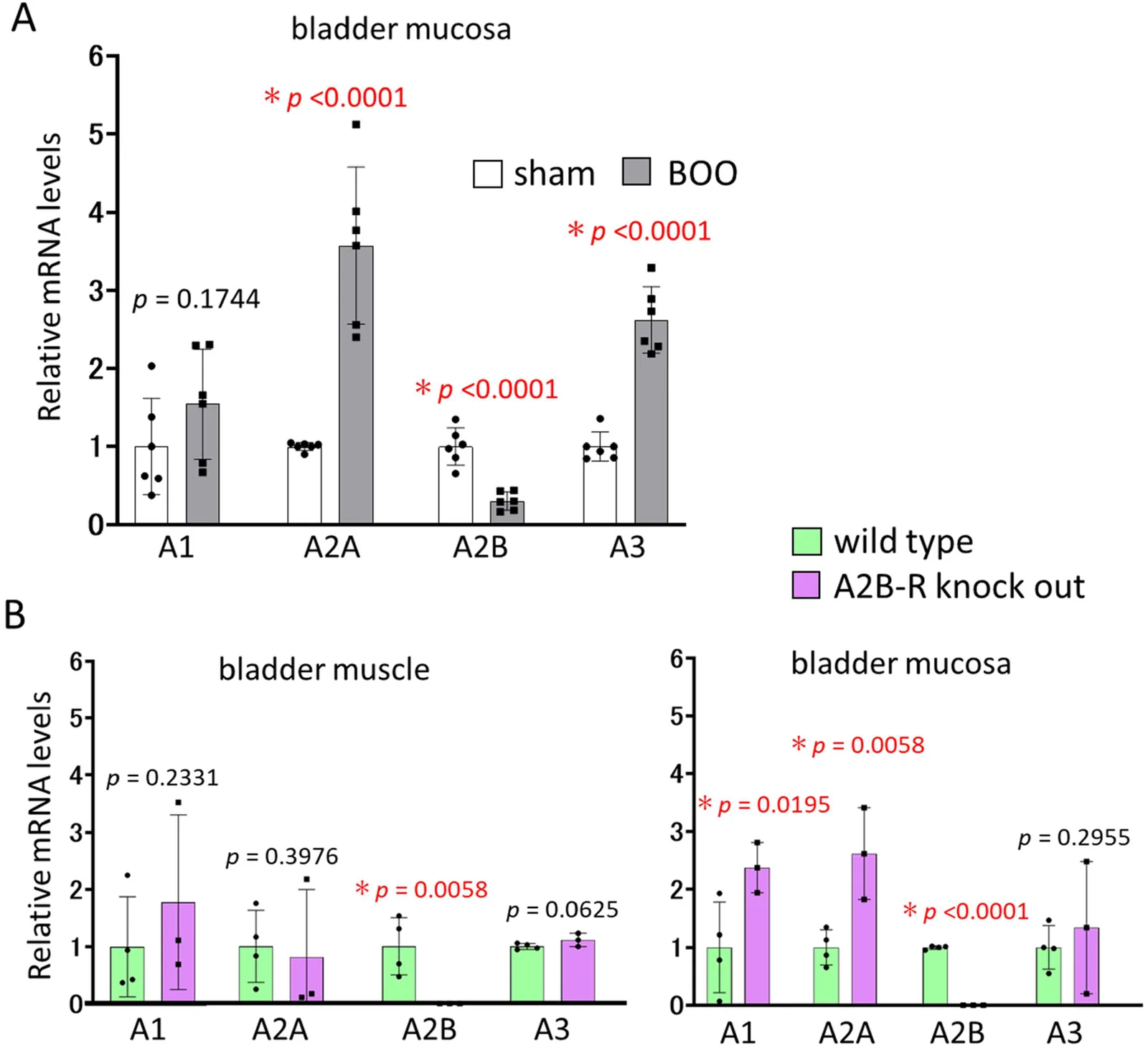
Relative mRNA expression levels of Adenosine receptor subtypes (A) Transcript levels in bladder mucosa between BOO and sham of Sprague-Dawley rats. (B) Transcript levels in bladder muscle and mucosa of wild-type Dahl rats and A2B knockout rats.

**TABLE 1 ∣:** Effects of intravesical administration of adenosine A3 receptor acting drugs on CMG parameters in BOO rats.

		Amplitude (cmH2O)	Bladder Capacity (ml)	Voiding Efficiency (%)	Compliance (ml/cmH_2_O)	NVC rate (NVC/min)
Vehicle		85.4 ± 18.3	1.96 ± 0.47	66.9 ± 14.7	0.283 ± 0.12	0.95 ± 0.26
A3 antagonist (MRS3777)	10 μM	84.9 ± 18.2	2.02 ± 0.43	67.6 ± 15.8	0.273 ± 0.04	1.43 ± 0.92
	100 μM	85.0 ± 17.2	1.98 ± 0.44	67.3 ± 16.2	0.232 ± 0.08	1.90 ± 0.82*
Vehicle		95.1 ± 17.8	1.78 ± 0.67	68.9 ± 11.8	0.231 ± 0.11	0.93 ± 0.28
A3 agonist (2CL-IB-MECA)	10 μM	95.2 ± 17.7	1.81 ± 0.62	68.9 ± 11.4	0.218 ± 0.10	0.83 ± 0.32
	100 μM	89.9 ± 15.9	1.74 ± 0.52	68.6 ± 9.42	0.206 ± 0.08	0.79 ± 0.40
Vehicle		85.5 ± 18.7	1.95 ± 0.44	66.7 ± 13.6	0.293 ± 0.09	0.95 ± 0.32
A3 antagonist 100 μM+ agonist 100 μM		86.1 ± 16.6	1.95 ± 0.24	66.3 ± 15.0	0.265 ± 0.06	0.96 ± 0.35

## Data Availability

The data that support the findings of this study are available from the corresponding author upon reasonable request.
