# Supplementary material for: Functional Roles of Adenosine A2B and A3 Receptor Subtypes in the Bladder of Male Rats With A2B Receptor Deletion or Bladder Outlet Obstruction
Source: Neurourol Urodyn. Author manuscript; Available in PMC 2026 Nov 3. (PMC13728411; doi:10.1002/nau.70430)
Supplement: Supplementary file 1 [file NIHMS2216795-supplement-Table_S1.pdf]

Supplementally Table S1. Primers for RT PCR

Gene name	Forward (5'→3')	Reverse (5'→3')	Reference
ADORA$_1$	GGATCGATACCTCCGAGTCA	GAGAATCCAGCAGCCAGCTA	Rose'Meyer RB, 2003
ADORA$_{2A}$	AAACACAGTGTGGCTCATTT	CAGGTCCCATGAGAGAGATTAG	Weller J, 2015
ADORA$_{2B}$	GCGTCCCGCTCAGGTATAAA	GTCCAATGCCAAAGGCAAGG	Haanes KA, 2014
ADORA$_{A3}$	CCCTCCCGCTTCATTTATTT	CCCTCAACGTTCACTCCATT	Weller J, 2015
GAPDH	GGCCAAAAGGGTCATCATCT	GTGATGGCATGGACTGTGGT	Yoshikawa S, 2017

Rose'Meyer RB, Mellick AS, Garnham BG, Harrison GJ, Massa HM, Griffiths LR.
The measurement of adenosine and estrogen receptor expression in rat brains following ovariectomy using quantitative PCR analysis.
Brain Res Brain Res Protoc. 2003 Mar;11(1):9-18.

Weller J, Pose M, Protzel C, Mader F, Porath K, Köhling R, Hakenberg OW, Kirschstein T.
Age-related decrease of adenosine-mediated relaxation in rat detrusor is a result of A2B receptor downregulation.
Int J Urol. 2015 Mar;22(3):322-9.

Haanes KA, Edvinsson L.
Expression and characterization of purinergic receptors in rat middle meningeal artery-potential role in migraine.
PLoS One. 2014 Sep 29;9(9):e108782.

Yoshikawa S, Sumino Y, Kwon J, Suzuki T, Kitta T, Miyazato M, Yoshimura N.
Effects of multiple simulated birth traumas on urethral continence function in rats.
Am J Physiol Renal Physiol. 2017 Nov 1;313(5):F1089-F1096
